# A Decision-Making Supporting Prediction Method for Breast Cancer Neoadjuvant Chemotherapy

**DOI:** 10.3389/fonc.2020.592556

**Published:** 2021-01-05

**Authors:** Dong Song, Xiaxia Man, Meng Jin, Qian Li, Han Wang, Ye Du

**Affiliations:** ^1^Department of Breast Surgery, The First Hospital, Jilin University, Changchun, China; ^2^Department of Oncological Gynecology, The First Hospital, Jilin University, Changchun, China; ^3^School of Information Science and Technology, Northeast Normal University, Changchun, China; ^4^Institute of Computational Biology, Northeast Normal University, Changchun, China

**Keywords:** breast cancer, treatment efficacy prediction, decision-making, multiple classification, neoadjuvant chemotherapy

## Abstract

Neoadjuvant chemotherapy (NAC) may increase the resection rate of breast cancer and shows promising effects on patient prognosis. It has become a necessary treatment choice and is widely used in the clinical setting. Benefitting from the clinical information obtained during NAC treatment, computational methods can improve decision-making by evaluating and predicting treatment responses using a multidisciplinary approach, as there are no uniformly accepted protocols for all institutions for adopting different treatment regiments. In this study, 166 Chinese breast cancer cases were collected from patients who received NAC treatment at the First Bethune Hospital of Jilin University. The Miller–Payne grading system was used to evaluate the treatment response. Four machine learning multiple classifiers were constructed to predict the treatment response against the 26 features extracted from the patients’ clinical data, including Random Forest (RF) model, Convolution Neural Network (CNN) model, Support Vector Machine (SVM) model, and Logistic Regression (LR) model, where the RF model achieved the best performance using our data. To allow a more general application, the models were reconstructed using only six selected features, and the RF model achieved the highest performance with 54.26% accuracy. This work can efficiently guide optimal treatment planning for breast cancer patients.

## Introduction

Breast cancer is the most frequent cancer in women. Diagnosed patients account for 30% of all female cancers (1), and the incidence rates continue to increase (2). Along with surgical treatment, neoadjuvant chemotherapy (NAC) has been introduced as a treatment strategy with the aim of reducing tumor size (3) and has contributed to significantly increase the efficacy of breast cancer treatment (4), thanks to its steadily increasing acceptance as a multidisciplinary treatment approach for patients with locally advanced breast cancer (5). NAC is a cost-efficient approach to downstage primary tumor and metastatic axillary lymph node (6), and has especially achieved notable pathologic complete response (pCR) rates in the most aggressive tumors (7), and conversely, also provides obvious benefits to breast-conservation therapy (8, 9). NAC takes advantage of and contributes to the impact of systemic therapies on breast cancer biology (10). However, the therapeutic effect of NAC relies on the patient’s biological phenotypes including HER2(+) positive cancers (11) or triple-negative cancers (12), which characterize the many patients who experience higher local recurrence rates (13).

Decision-making therefore is a vitally important part of NAC, and treatment regiments selected according to the patients’ individual needs will help to optimize NAC, in which a comprehensive treatment evaluation is required. Undoubtedly, a clinical evaluation (14–16), tissue pathology evaluation (17, 18), evaluation of axillary lymph nodes (19), and a combination of these evaluation approaches (20) will accurately contribute to the efficacy of NAC, although all these evaluations are posterior approaches, regardless of the outcome of treatment given to a designated patient. Therefore, prospective treatment effects may be likely foreseen for patients, who will benefit from the selection of an optimal treatment regiments, that avoids adverse events, and thereby reduces the recurrence, metastasis, and latent risks.

Many computational methods have been developed to predict the survival of breast cancer patients receiving NAC treatment based on the information recorded during the clinical treatment processes. Recently, Lai et al. proposed a prognostic nomogram model to predict disease-free survival (DFS) (21), Laas et al. used a random survival forest method to evaluate distant metastasis-free survival (DMFS) (22), and Tahmassebic et al. compared eight machine learning algorithms for the early prediction of pCR, including the support vector machine (SVM), linear discriminant analysis (LDA), extreme gradient boosting (XGBoost), where the XGBoost algorithms attained the best performance (23). The above methods achieved good prediction accuracy, and demonstrated that the computational approach is a practical contribution to evaluating NAC. Cancer survival is determined by many factors, and sample quantity seems less than the requirement of machine learning algorithms to make a comprehensive regression prediction at present. By contrast, grading evaluation may even more intuitive to guide the decision-making of the treatment.

In this study, we adopted the Miller-Payne grading system to evaluate the efficacy of NAC, the clinical treatment records of 166 Chinese breast cancer cases from the First Bethune Hospital of Jilin University were used to build the evaluation model. The 166 cases were randomly selected as the training dataset. For the comparison, three traditional machine learning algorithms, random forest (RF), logistic regression (LR), and SVM were used, along with the CNN deep learning method. The four models were trained and tested using the same datasets, and the RF model achieved the best performance after feature selection. This work merely uses basic clinical data to accurately evaluate NAC efficacy, it represents a promising approach to improve the treatment decision-making process.

## Materials and Methods

### Patients and Treatment Regiments

A total of 166 breast cancer cases were collected among the diagnosed patients at the First Bethune Hospital of Jilin University over the past five years, which included triple-negative, Luminal A, HER2 positive, Luminal B(+), Luminal B(-) cases, covering the patients aged 27 to 71 years old. These pathological classifications are molecular subtypes of breast cancer and are used to predict the risk of recurrence and metastasis of breast cancer and its response to treatment. All patients received surgical treatment based on their diagnosed cancer types, which consisted of radical mastectomy, protective and radical operation of mastocarcinoma, and breast conserving surgery. Correspondingly, the NAC regiments were differentially applied to individual patients, while chemotherapy was based on an anthracycline and paclitaxel chemotherapy regimen, and Herceptin-targeted therapy was given to HER2 positive patients before chemotherapy. All NAC patients received radiotherapy, and endocrine therapy was adopted after radiotherapy.

The diagnosis and NAC treatment process were recorded for each patient. All data were digitalized and cleaned, record items having too many default values were filtered, and otherwise missing values were assigned to zero. Finally, 26 clinical record items were available as features to be input into the prediction model. Detailed information regarding the selected features is shown in **Supplementary Table 1**. However, not all features contributed to obtaining an accurate prediction, we thus selected only 6 features that were highly relevant to the Miller-Payne grading scale, including Ki-67 expression, breast mass length, breast mass width, PR value, visible tumor thrombi, final calcification morphology. For the experimental study, 166 cases were divided into a training dataset based on 10-fold cross-validation.

### NAC Evaluation

The Miller-Payne grading system (24) provides a five-point scale by the paired examination of specimens before and after the operation, and is currently widely applied in clinical treatment evaluation in China, including for NAC evaluation in this study. Based on the proportion of tumor cell reduction, the system grades the postoperative curative effect from level 1 to 5 according to the decrease in tumor cells from low to high. This system does not provide a comprehensive evaluation of postoperative cancer survival investigations, but it is obvious that there are few evaluation approaches available for this purpose, as multiple factors are involved in cancer patient survival. In contrast, this system describes the principle pathological features relevant to cancer simply and intuitively, so that it is available for our goal to predict the NAC efficacy, and provides the suggestions for clinical practice.

### Feature Selection

The Pearson correlation coefficient (25) and the Random Forest Feature Importance Index (RFFII) (26) were used to select features that had the greatest impact on the model performance. Too many features may weaken the performance of the model, thus feature selection should be used to improve the model’s performance. PCC can identify the linear relationship between features and labels while the RRFII selects the feature importance relative to the RF prediction model. We combined these indicators as a reference for feature selection. The PCC was used to calculate the linear relationship between each feature and the Miller-Payne grading label. It divided feature correlation into positive and negative categories, in which the higher the absolute value of the correlation coefficient the stronger the correlation.

The RFFII presents the degree to which each feature contributes to each tree in a random forest, then takes the average value, and finally compares the contribution of different features. For example, for each decision tree in a random forest with N trees, the corresponding Out-of-Bag (OOB) data was used to calculate its OOB error, which is recorded as e^1^. Then, noise interference is randomly added to the features of OOB samples, and the OOB is calculated again, which is then recorded as e^2^. The characteristic importance D of the whole random forest can be calculated using Eq. (1).

(1) $$D=\sum \frac{e^{2}-e^{1}}{N}$$

### Prediction Models

For Miller-Payne grading, a multiple classifier was applied to predict NAC efficacy; there are many choices of algorithms available to establish the prediction model. In previous studies, the Scikit-Learn (Sklearn) classifier was widely used as a machine learning tool based on Python, and provides various packaged tools. Among the numerous machine learning algorithms, we choose to apply RF (27, 28), LG (29), and SVM (30) algorithms for modeling. Further, a CNN (31) model was built based on Keras since the deep learning module delivered good performance in many similar studies. In total four algorithms were attempted in this study to build the prediction models, and the best model was selected to be used in the clinical setting.

#### RF Model

RF is a modified algorithm based on bagging strategy, using multiple trees to train and classify the input sample, it builds each tree according to the following algorithm:

1. Draw a bootstrap sample Z* of size N from the training data
2. Grow a RF tree T to the bootstrapped data, by recursively repeating the following steps for each terminal node of the tree, until the minimum node size n is reached.
  (1) Select m variables at random from the M variables
  (2) Pick the best variable among the m
  (3) Split the node into two daughter nodes
3. Output the ensemble of trees

N was used to represent the number of training cases (samples), and M was used to represent the number of features. In this study, there were 166 training cases with 26 features, the M is equal to 26 and the N is equal to 166.

The default parameters were used to initialize the model, and then we tuned the parameters to adjust the model to achieve the best performance. There were three parameters to be optimized, including the optimal minimum sample number of leaf nodes (min_samples_leaf), the number of trees (n_estimators), and the minimum sample size required for internal node repartition (min_samples_split). The min_samples_split value limits the conditions under which subtrees can continue to be divided, and if the sample size of a node is less than the min_samples_split, it will not continue to attempt to select the optimal feature for partitioning. The min_samples_leaf was 3 and the n_estimators was 400 after parameter tuning. The min_samples_split was set to 2 to limit the minimum sample size.

#### LG Model

Logistic regression is a machine learning method used to solve binary classification (0 or 1) problems. It is often used to estimate the possibility of something. LG model transformed the 26 features into a virtual variable as the input variable. The model executed data analysis and established a decision boundary. Subsequently, using the Sigmoid function and the gradient descent were used to solve the optimization problem. The optimal solution was obtained and output as the Miller-Payne grades.

#### SVM Model

SVM is a binary classification model. Its basic model is a linear classifier with the largest interval defined in the feature space. Using 26 features as the input variable of SVM model. The SVM model attempts to find a suitable classification hyperplane to classify the data, which is defined as Eq. (2). The SVM model can obtain more than one hyperplane, so loss of function optimization is used to identify the classification hyperplane with the strongest generalization ability in order to achieve the classification effect and build a multiple classifier. Next, the Millar-Payne grades were output.

(2) $$w^{T}x+b=0$$

#### CNN Model

CNN is a type of feedforward neural network that contains convolution calculation and has a deep structure. It can represent learning ability and classifies input information according to its hierarchical structure. Twenty-six features were input into the CNN model through the input layer. Then the convolution kernel was used in the convolution layer to extract and map the features. Features were obtained by multi-layer convolution and then they were classified in the full connection layer. In order to prevent overfitting, a pooling operation was carried out, and the features were aggregated to reduce the amount of data operation. The ReLU function was used as the activation function to increase the nonlinear relationship among the layers of the neural network. In this study, five full connection layers were used, and the nodes of each layer were set as 2,048, 1,024, 564, 256, and 6. The Miller-Payne grades were output in the last full connection layer.

The input of these four models included the 26 features of the NAC dataset. The known labels cases were used to train the model while the test cases that were unknown labels were used to test the model. Finally, the output obtained represented the predicted labels of unlearned test cases. we then evaluated the performance of these models and chose the best-performing machine learning model for our prediction model.

### Training and Evaluation

For the purpose of the model training and prediction performance evaluation, we used 10-fold cross-validation to train the models and evaluated them by Accuracy (ACC), Precision (P), F1 score (F1), and Recall (R). A 10-fold cross-validation is a common training and validating method, it randomly divides the dataset into ten subsets, each turn of total ten in the validation process, chooses one subset as the testing dataset, and the remaining nine are the training dataset (32, 33). The correct rate (or error rate) was obtained for each test. The average value of the accuracy (or error rate) of the 10 times results was used as the estimation of the accuracy of the algorithm.

The weighted average value was applied to evaluate the performance of the model more accurately. The sample quantity of Miller-Payne grading was uneven, for example, there were only two samples in the first category. An uneven distribution of samples will affect the performance of the prediction model. The value range is from 0 to 1.

ACC is the evaluation of the overall accuracy of classification, so a calculation of the weighted average is not needed. It was calculated using Eq. (3). Precision was defined by evaluating the accuracy of each category to the classifier. It was calculated using Eq. (4). Recall is a supplement to precision and the recall rate was obtained for our original sample, which indicated the number of positive examples in the sample were predicted to be correct. It was calculate using Eq. (5). The F1-score represented the harmonic average of precision and recall. It was calculated using Eq. (6).

(3) $$ACC=\frac{TP+TN}{TP+TN+FP+FN}$$

(4) $$P=\frac{TP}{TP+FP}$$

(5) $$R=\frac{TP}{TP+FN}$$

(6) $$F1=\frac{2*P*R}{P+R}$$

## Results and Discussion

### Prediction Performances on Original Features

In this study, we used the dataset collected from patients diagnosed at the First Bethune Hospital of Jilin University over the past three years. Patients were divided into triple-negative, Luminal A, HER2(+), Luminal B(+), Luminal B(-) types, and involved patients aged from 27 to 71 years old. The dataset including 166 clinical cases. LR, SVM, CNN, and RF were used to construct prediction models of the Miller-Payne grading system for the NAC regiment. The original dataset obtained 26 features that were used as the input features for the models and a 10-fold cross-validation was used to train these models and ACC, P, F1, and R scores were used to evaluate their performance.

The evaluation results of the RF, LG, SVM, and CNN models are shown in **Table 1**. The ACC scores of the RF, LG, and SVM models were 49.45, 48.24, and 50.00%, respectively. The CNN model achieved the best ACC score at 50% and the SVM model achieved the worst ACC score at 45.84%. The LG model had the best scores in P, F1, and R score which was 49.72, 50.00, and 49.72%, respectively, while the SVM model had the worst scores which were 45.39, 43.37, and 44.13%, respectively. The evaluation scores of level 1 were zero, which could be due the fact that there were only 2 samples in level 1 of the Miller-Payne grading in the dataset, and thus, the prediction model could not be trained. From these scores it can be derived that the performance of these machine learning models did not perform very well for this dataset, and the overall evaluation score of these four models did not differ greatly. This might be due to model overfitting caused by the redundant features that affect the performance of the models. CNN achieved the best score, which may have resulted from the pooling operations in the layers of the neural network to reduce overfitting. Therefore, feature selection is required. We thus compared the prediction performance of models using the original features and selected the original features using the PCC and RFFII algorithms.

**Table 1:** Prediction Performances on Original Features.

		Level 1	Level 2	Level 3	Level 4	Level 5	Weighted	Accuracy
	Amount	2	41	50	43	30		
RF	Precision	0	50.00%	48.39%	46.94%	54.55%	48.94%	49.45%
	Recall	0	26.83%	60.00%	53.49%	60.00%	49.40%	
	F1-score	0	34.92%	53.57%	50.00%	57.14%	48.04%	
LG	Precision	0	**54.05%**	50.94%	48.98%	46.15%	**49.72%**	48.24%
	Recall	0	**48.78%**	54.00%	**55.81%**	40.00%	**50.00%**	
	F1-score	0	**51.28%**	52.43%	**52.17%**	42.86%	**49.72%**	
SVM	Precision	0	37.14%	**59.52%**	43.48%	38.89%	45.39%	45.83%
	Recall	0	31.71%	50.00%	46.51%	46.67%	43.37%	
	F1-score	0	34.21%	54.35%	44.94%	42.42%	44.13%	
CNN	Precision	0	25.00%	46.67%	**54.55%**	**58.33%**	45.85%	**50.00%**
	Recall	0	11.11%	**70.00%**	46.15%	**77.78%**	**50.00%**	
	F1-score	0	15.38%	**56.00%**	50.00%	**66.67%**	46.39%	

Bold values represent the maximum values of precision, recall, F1-score and accuracy among the four machine learning models.

### NAC Efficacy Relevant Features

For clinical purposes, the principal features would be more efficient as tools for making treatment-related decisions, to reduce the computational costs, or even to improve prediction accuracy. We performed feature selection to identify the principle features from the total of 26 original features input by PCC and RFFII algorithms.

**Figure 1:**
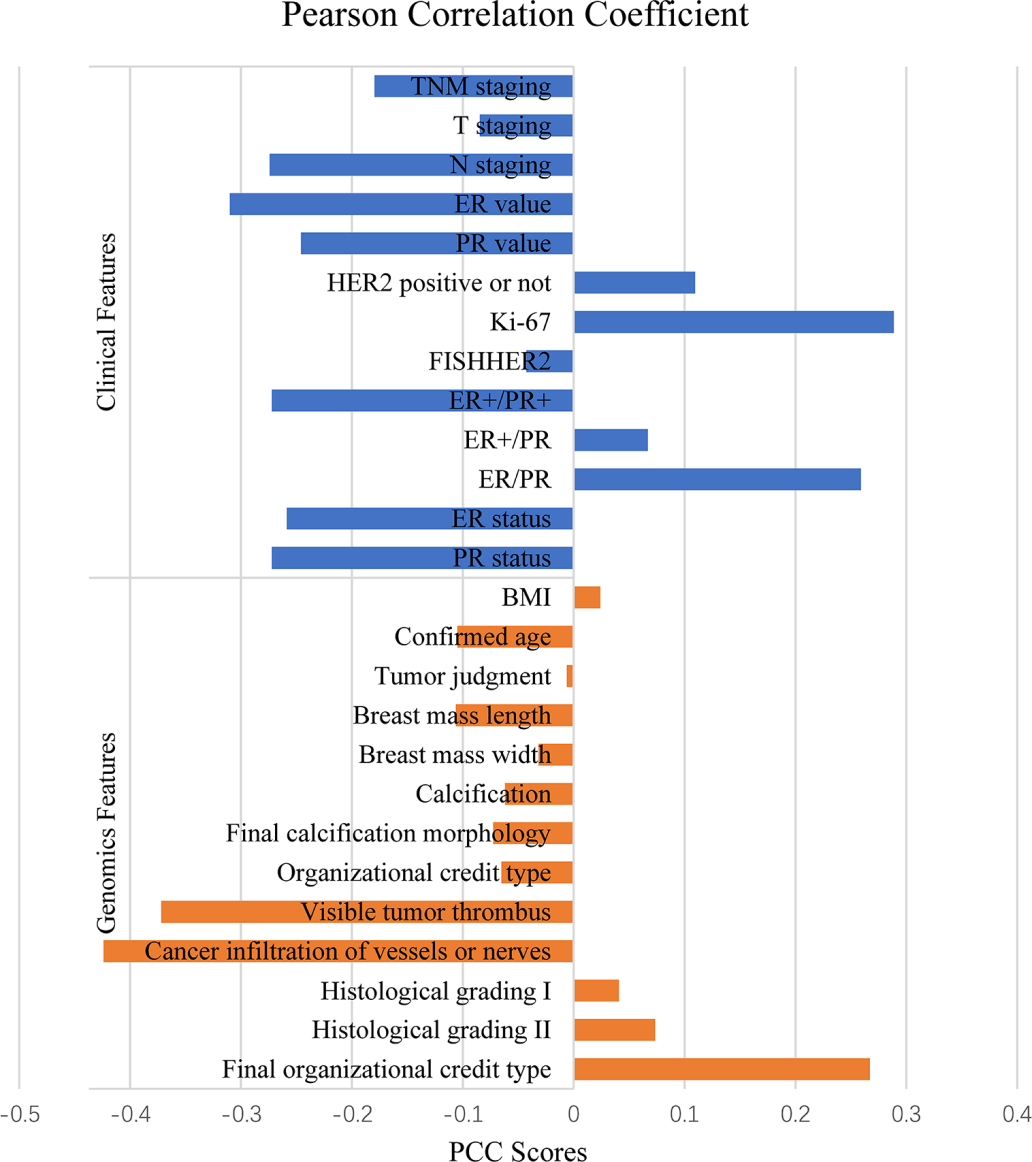
Features relevant to NAC by Pearson correlation coefficient.

PCC was applied to calculate the correlation between Miller-Payne grading and each feature for all the 166 cases and then ranked the correlation coefficients according to their absolute values. There were 8 features positively correlated to Miller-Payne grading while 18 features were negatively correlated. As shown in **Figure 1**, Ki-67 expression, final organizational credit type, ER/PR status were positively correlated, while cancer infiltration of vessels or nerves, visible tumor thrombus, and ER value negatively correlated; Ki-67 expression, ER/PR and ER values represented genomic features. Ki-67 expression is associated with prognosis in breast cancer patients, which helps to determine whether or not NAC should be applied. The ER/PR and ER values can also be used to predict prognosis of breast cancer patients and guide the NAC treatment option. Final organizational credit type, cancer infiltration of vessels or nerves, and visible tumor thrombus are used to judge the condition of patients before NAC is attempted.

**Figure 2:**
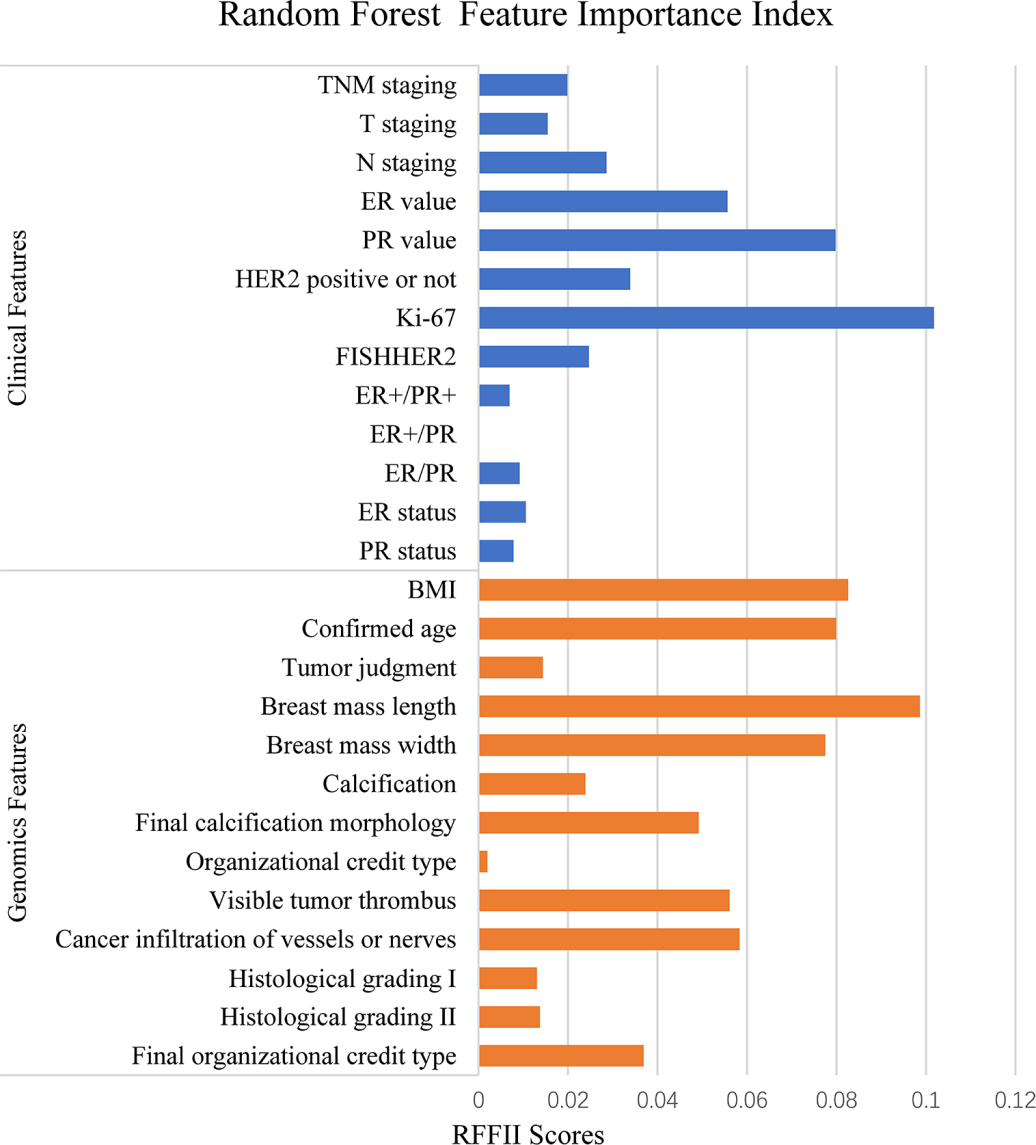
Features related to NAC by Random Forest correlation.

We then classified the clinical features into positive correlation features and negative correlation features according to PCC, and ranked these according to absolute values. Similarly, we used the RFFII to rank the features. Among positive correlation and negative correlation features, features with a higher correlation index were selected as the input features to train the model. The features with higher PCC and RFFII scores were input as priority. As shown in **Figure 2**, the abscissa indicates the RFFII score; the higher the score obtained by the feature, the greater the importance in the RF model. Ki-67 expression, PR and ER values are important genomic features, while breast mass and width, visible tumor thrombus, final calcification morphology, and the body mass index are important clinical features.

**Figure 3:**
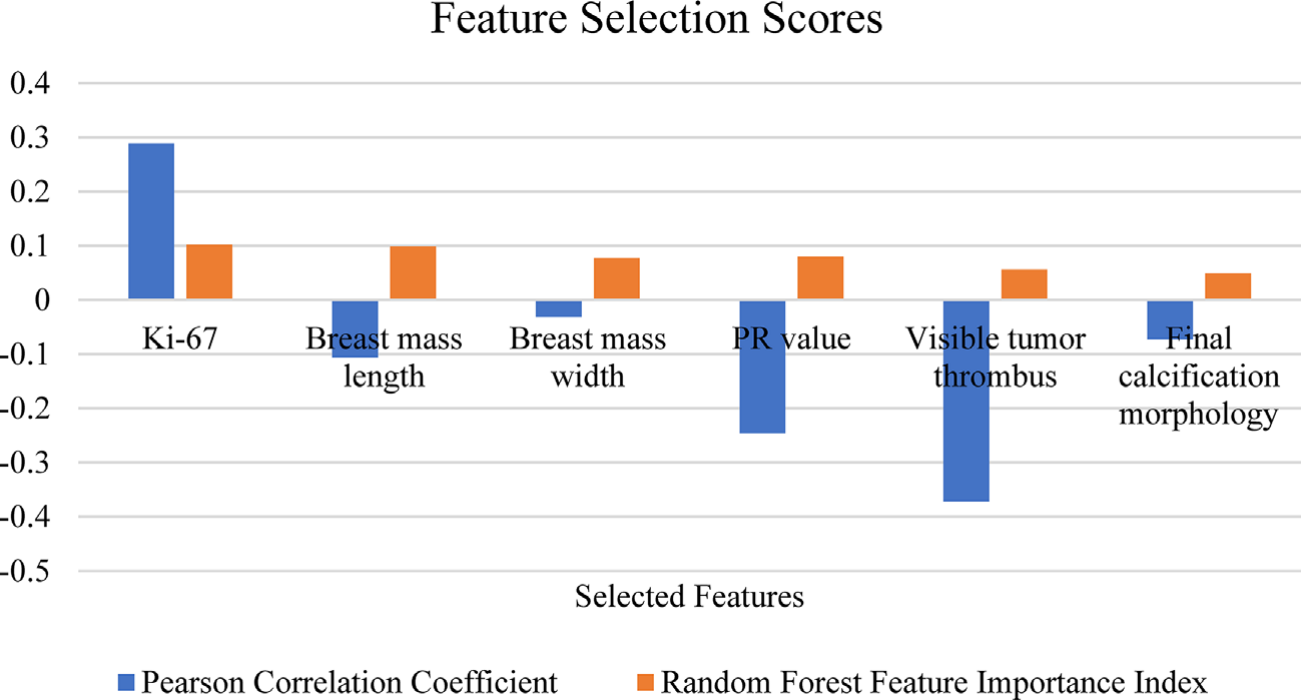
Selected feature attributions.

In the feature selection process, each group of input features was input into four models for training, and the performance of each model was evaluated. According to the two indicators, the features were iterated from high to low as input to train the models. Features that had a bad impact on the model performance were eliminated and finally a group of features with the highest accuracy rate was selected. Finally, the best performing model was selected as the final prediction model and the group of features was selected. The performance of the RF model was the best and included an input group comprising six features: Ki-67, breast mass length, breast mass width, PR value, visible tumor thrombus, final calcification morphology (**Table 2**). **Figure 3** shows the PCC and RF correlation of these 6 characteristics. The Ki-67 expression and PR value are genomic features and were positively related to the Miller-Payne grading, and showed a high correlation with RF. Breast mass length, breast mass width, visible tumor thrombus, and final calcification morphology were the clinical features having high PCC and RFFII scores, and produced a great influence on the prediction model.

**Table 2:** Selected Features.

Feature label	Numerical meaning
Ki-67	The higher the index, the more tumor cells are proliferating and the higher the malignant process is
Breast mass width	Width of breast tumor
Breast mass length	Length of breast tumor
PR value	Progesterone receptor value
Visible tumor thrombus	Class 0: No visible tumor thrombus
	Class 1: Tumor thrombus can be seen in vessels
	Class 2: Tumor thrombus can be seen in the nerve
	Class 3: The vessels and nerves were all visible
Final calcification morphology	Class 1: Punctate calcification
	Class 2: Cluster calcification
	Class 3: Minute calcification
	Class 4: Lineal calcification

### Prediction Performances on Selected Features

In the feature screening stage, the RF model was identified as the final prediction model. We retrained the other three models and evaluated their performance in comparison with the RF model using the features after feature selection.

**Table 3** shows the evaluation results of the RF, LG, SVM, and CNN models after feature selection, and reports the respective ACC scores 54.26, 47.62, 39.81, and 47.62% for each model. The RF model obtained the highest ACC, P, F1, R scores. The performance of RF model significantly improved as a whole, while the performances of the LG, SVM, and CNN models declined.

**Table 3:** Prediction Performances on Selected Features.

		Level 1	Level 2	Level 3	Level 4	Level 5	Weighted avg	Accuracy
RF	Precision	0	**53.57%**	52.46%	**48.94%**	**66.67%**	**53.76%**	**54.26%**
	Recall	0	**36.59%**	64.00%	53.49%	**66.67%**	**54.22%**	
	F1-score	0	**43.48%**	**57.66%**	**51.11%**	**66.67%**	**53.39%**	
LG	Precision	0	50.00%	**52.83%**	45.83%	45.71%	48.40%	47.62%
	Recall	0	**36.59%**	56.00%	51.16%	53.33%	48.40%	
	F1-score	0	42.25%	54.37%	48.35%	49.23%	48.23%	
SVM	Precision	0	42.11%	41.18%	41.67%	40.43%	40.90%	39.81%
	Recall	0	19.51%	**70.00%**	11.63%	63.33%	40.36%	
	F1-score	0	26.67%	51.85%	18.18%	49.35%	35.83%	
CNN	Precision	0	11.11%	35.71%	46.67%	100.00%	46.76%	47.62%
	Recall	0	11.11%	50.00%	**53.85%**	44.44%	40.48%	
	F1-score	0	11.11%	41.67%	50.00%	61.54%	40.96%	

Bold values represent the maximum values of precision, recall, F1-score and accuracy among the four machine learning models.

The RF model achieved the best score overall, thus we selected the RF model as the prediction model to predict the NAC outcomes. Our selection was supported by consulting the RFFII index in the feature selection module. The application of the index makes allows the RF model to eliminate some redundant features. Our original dataset contained many clinical and genomic features, while in the initial training stage, the models could not distinguish which features were associated with Miller-Payne grading. In the training process, the RF modelling generated different decision trees according to the importance of the single features and then output the optimal solution. Therefore, after removing redundant features through feature selection, the performance of the RF model was significantly improved.

**Figure 4:**
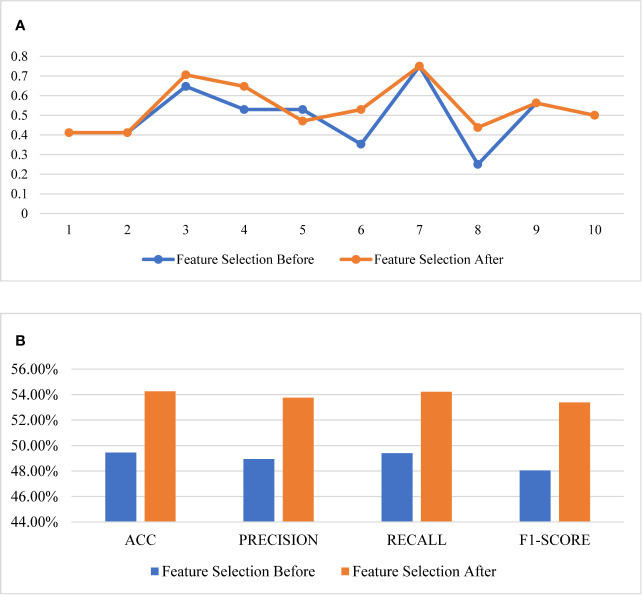
Comparison of the performance of feature selection. **(A)** The 10-fold cross validation results, **(B)** the scores of the models.

The comparison of the evaluation curves of the 10-fold cross-validation between the RF models for the original 26 features and the selected 6 features is shown in **Figure 4**. The score of 10-fold cross validation after feature selection generally rose, and the performance of the prediction model improved substantially after feature selection. The model before feature selection achieved 49.45% of the ACC score, 48.94% of the precision score, 49.40% of the recall score, and 48.04% of the f1-score. The same model after feature selection achieved 54.26% of the ACC score, 53.76% of the precision score, 54.22% of the recall score, and 53.39% of f1-score.

### Case Studies

In order to represent the prediction ability, we randomly selected 4 cases from the four major categories of Miller-Payne grading in our dataset, then 16 cases were input into the final prediction model as an evaluation set, the corresponding results are listed in **Table 4**. Fourteen of the 16 cases were correctly predicted, while case 5 was mispredicted as grade level 4 instead of level 3, and case 14 as level 5 instead of its true level 4. We will analyze these two cases in detail. First, the feature PCC scores were calculated for each of the cases using the 4 representative cases indicated above in its truth category, and then using the averaged PCC score was used to compare predicted values. In the resulting output of the final predictions, case 5 obtained a score of 0.4347 in its truth grade level 3, but merely a score of 0.3591 in the predicted grade level 4, a result which appeared to indicate that case 5 was more similar to cases from level 3 in with regard to the clinical data features. In contrast, case 14 was very similar to cases in level 4 as we predicted, where the score value was 0.8969 compared to a score of 0.8539 in the truth grade level 5.

**Table 4:** Case Details.

Input Case	1	2	3	4	5	6	7	8	9	10	11	12	13	14	15	16
Ki-67	2	2	2	2	3	3	3	3	4	4	4	4	5	5	5	5
Breast mass length	2	30	10	25	3	60	30	30	0	10	0	20	70	30	70	85
PR value	2.9	5	3	3.2	4.7	4.3	1.45	3.45	1	2	3.5	1.58	0.8	2.9	4.84	4.8
Breast mass width	3	60	80	10	5	0	80	80	1	11	0	70	0	0	0	0
Visible tumor thrombus	1.3	2.3	1.4	2	2.8	2	0.84	1.6	1	1.5	3	1.32	0.5	2	1.33	3
Final calcification morphology	0	2	4	0	2	0	0	8	2	2	3	0	1	7	1	8
Miller Payne grading	2	2	2	2	**3**	3	3	3	4	4	4	4	5	**5**	5	5
Forecast results	2	2	2	2	**4**	3	3	3	4	4	4	4	5	**4**	5	5

Bold values represent the cases which were mis-predicted by the model.

Although the PPC scores of the individual features could not be used alone to determine a predicted value for each case, there were also obvious differences among cases in the same grade category, which is a common phenomenon in the clinical diagnostic setting. The latter is an indication supporting the application of machine learning models, which are able to discover inner laws from unintuitive clinical data. Furthermore, from cases 5 and 14, the modelling outcomes suggest that no one approach is able to comprehensively evaluate NAC efficacy, there may be some “fuzzy” zones in the evaluation similar to those observed in the Miller-Payne grading system. Consequently, the duty of the corresponding prediction methods is to assist, but not make the definitive clinical treatment decisions, namely, the predictions of outcomes should be made to approach the true outcome but should be accompanied by the accumulated clinical data for each case.

## Conclusion

We propose a practical breast cancer treatment efficacy prediction tool for NAC patients. Twenty-six features were extracted from 166 cases of real-life clinical data provided by the First Bethune Hospital of Jilin University, and ultimately 6 principle features were selected using a feature selection process, which were finally used to optimally construct the supervised prediction model using the Miller-Payne grading system as a NAC treatment evaluation tool. Four different machine learning methods were testing in the study, of which the Random Forest model proved to be the most compatible with an average accuracy of 54.26%. As discussed in the case studies, accuracy could not be considered a comprehensive criterion in the clinical application given the complexity of each case. Nonetheless, our method efficiently predicted outcomes of all the cases that were similar to their true grade. Our findings suggest this approach may provide an important contribution to the decision-making process for the clinical treatment of breast cancer.

## Data Availability Statement

Requests to access the datasets should be directed to DS, songdong690117@163.com.

## Ethics Statement

The studies involving human participants were reviewed and approved by Medical Ethics Committee of the First Hospital of Jilin University. Written informed consent for participation was not required for this study in accordance with the national legislation and the institutional requirements.

## Author Contributions

DS, YD, XM, and QL contributed to the conception of the study, clinical data collection, and the efforts of the clinical application. HW and MJ constructed the prediction models and all the relevant computational experiments. All authors contributed to the article and approved the submitted version.

## Funding

This work was supported by the National Natural Science Foundation of China (No. 81773171), the Science and Technology Department of Jilin Province (Nos. 20170311005YY, 20200404197YY, 20200201349JC, and 20180414006GH, and Fundamental Research Funds for the Central Universities (No. 2412019FZ052).

## Conflict of Interest

The authors declare that the research was conducted in the absence of any commercial or financial relationships that could be construed as a potential conflict of interest.
