# Supplementary material for: A Decision-Making Supporting Prediction Method for Breast Cancer Neoadjuvant Chemotherapy
Source: Front Oncol. 2021 Jan 5;10:592556. doi: 10.3389/fonc.2020.592556 (PMC7813988; doi:10.3389/fonc.2020.592556)
Supplement: Supplementary file 1 [file Table_1.docx]

Supplementary Material

# Supplementary Figures and Tables

## Supplementary Table

**Supplementary Table 1.** Features information. Using numbers to represent the level of the features.

| Feature label | Numerical meaning | |
| --- | --- | --- |
| Ki-67 | The higher the index is, the more tumor cells are proliferating and the hig | |
|  | her the malignant process is | |
| Visible tumor thrombus | Class 0: No visible tumor thrombus | |
|  | Class 1: Tumor thrombus can be seen in vessels | |
|  | Class 2: Tumor thrombus can be seen in the nerve | |
|  | Class 3: The vessels and nerves were all visible | |
| N staging | Class 1: Lymphoma was distributed on one side of the axis symmetry line | |
|  | Class 2: Lymphoma was distributed on both sides of the axis symmetry line | |
|  | Class 3: Lymphoma is located on both sides of mediastinum | |
|  | Class 4: Lymphoma is widely distributed and invades other regions | |
| TNM staging | Class 0: Tis N0 M0 | |
|  | Class 1: T1 N0 M0 | |
|  | Class 2: | T0 N1 M0 |
|  |  | T1 N1 M0 |
|  |  | T2 N0 M0 |
|  | Class 3: | T2 N1 M0 |
|  |  | T3 N0 M0 |
|  | Class 4: | T0 N2 M0 |
|  |  | T1 N2 M0 |
|  |  | T2 N2 M0 |
|  |  | T3 N1 M0 |
|  |  | T3 N2 M0 |
|  | Class 5: T4 N0-N2 M0 | |
|  | Class 6: Any T N3 M0 | |
|  | Class 7: Any T any N M1 | |
| Histological grading І | Class 0: Differentiation unknown | |
|  | Class 1: High differentiation | |
|  | Class 2: Medium differentiation | |
|  | Class 3: Low differentiation | |
| Calcification | Class 0: no calcification | |
|  | Class 1: calcification | |
| Confirmed age | Age of breast cancer diagnosed of patients | |
| Final organizational credit type | Class 1: Luminal A type | |
|  | Class 2: Luminal B HER2 (-) type | |
|  | Class 3: Luminal B HER2 (+) type | |
|  | Class 4: HER2 overexpression type | |
|  | Class 5: Triple negative type | |
| ER/PR | Class 0: Negative | |
|  | Class 1: Positive | |
| HER2 positive or not | Class 0: Negative | |
|  | Class 1: Positive | |
| Histological grading II | Class 0: Unknow the grade | |
|  | Class 1: No more than grade III | |
|  | Class 2: Grade III | |
| ER+/PR | Class 0: Negative | |
|  | Class 1: Positive | |
| BMI | Body mass index | |
| Tumor judgment | Class 1: Left breast CA | |
|  | Class 2: Right breast CA | |
| Breast mass width | Width of breast tumor | |
| Breast mass length | Length of breast tumor | |
| FISHHER2 | Class 0: The threshold was classified as negative | |
|  | Class 1: The gene was amplified | |
|  | Class 2: No amplification of the gene | |
|  | Class 3: Unknown | |
| Organizational credit type | Class 1: Invasive ductal carcinoma | |
|  | Class 2: Invasive lobular carcinoma | |
|  | Class 3: Mucinous carcinoma | |
|  | Class 4: Invasive ductal carcinoma with | |
| Final calcification morphology | Class 1: Punctate calcification | |
|  | Class 2: Cluster calcification | |
|  | Class 3: Minute calcification | |
|  | Class 4: Lineal calcification | |
| T staging | Class 1: χ≤2cm | |
|  | Class 2: 2cm＜χ≤5cm | |
|  | Class 3: χ＞5cm | |
| PR value | PR value | |
| ER status | Class 0: Negative | |
|  | Class 1: Positive | |
| PR status | Class 0: Negative | |
|  | Class 1: Positive | |
| ER+/PR+ | Class 0: Negative | |
|  | Class 1: Positive | |
| ER value | ER value | |
| Cancer infiltration of vessels or nerves | Class 0: Negative | |
|  | Class 1: Positive | |
